# Supplementary material for: A Next-Generation Sequencing-Based Approach to Identify Genetic Determinants of Antibiotic Resistance in Cambodian Helicobacter pylori Clinical Isolates
Source: J Clin Med. 2019 Jun 15;8(6):858. doi: 10.3390/jcm8060858 (PMC6617194; doi:10.3390/jcm8060858)
Supplement: Supplementary file 1 [file jcm-08-00858-s001.pdf]

Table S1. Detailed information of 55 *H. pylori* strains in Cambodia.

Strains Name	Number of Contigs	Total Length [a]	Largest Contig [a]	Total Length [b]	GC [b] (%)	N50 [b]	N75 [b]	L50 [b]	L75 [b]
KH0001	50	1588767	211696	1586073	38.95	66557	46968	7	14
KH0012	48	1581240	236728	1578511	38.84	68881	48380	7	14
KH0016	43	1609166	150538	1608358	38.77	65730	48416	8	15
KH0022	43	1622236	385645	1619790	38.66	100932	56619	4	10
KH0025	39	1632223	312085	1630856	38.88	110306	46581	4	11
KH0031	31	1561234	296259	1560002	38.94	79421	53122	5	12
KH0032	47	1593989	179899	1592332	38.84	67583	42257	8	15
KH0033	69	1630094	180870	1626564	38.69	47133	32018	11	22
KH0040	52	1620336	287366	1617881	38.86	60039	39139	6	14
KH0042	48	1615198	233143	1611248	38.71	87895	54127	6	12
KH0045	47	1707464	299607	1704416	38.66	100584	53366	6	12
KH0054	66	1646489	237218	1642923	38.77	52684	33009	8	17
KH0065	51	1649404	327643	1647719	38.76	68849	41684	6	14
KH0066	46	1564277	178812	1562983	38.88	63714	50066	9	16
KH0070	32	1650045	202233	1649179	38.78	84470	64501	7	13
KH0071	40	1643911	171520	1643911	38.75	80136	52843	7	13
KH0072	36	1656729	271054	1655494	38.7	109937	47317	5	11
KH0075	53	1609845	235863	1607795	38.68	66619	47419	7	15
KH0086	41	1588593	133959	1587421	38.82	104540	52601	7	14
KH0092	45	1635651	240758	1634803	38.8	79342	43094	7	13
KH0095	42	1646965	311956	1646193	38.62	73496	49245	7	14
KH0097	49	1598890	202325	1597301	38.97	64907	44264	8	15
KH0106	42	1628359	228884	1627664	38.7	80488	56476	7	13
KH0110	33	1601682	213827	1601682	38.66	83106	54524	6	12
KH0113	32	1605875	240869	1605544	38.74	78049	48004	6	12
KH0119	43	1599435	230210	1597549	38.99	80905	52373	7	13
KH0120	51	1595648	235541	1592316	38.77	58574	38235	7	15
KH0122	55	1598940	179056	1598458	38.95	63441	34169	8	17
KH0125	51	1598142	185737	1596024	38.94	66891	34557	8	16
KH0126	49	1646157	199914	1644359	38.72	98970	36658	6	14
KH0127	25	1572859	234216	1572577	38.95	153258	67615	5	9
KH0129	48	1590246	173889	1588894	38.96	67221	47755	8	15

KH0141	36	1600673	244028	1600673	38.95	79605	50976	6	13
KH0143	59	1581211	127539	1580096	38.89	58076	36768	9	18
KH0148	42	1586701	238933	1585233	38.89	68938	52644	6	12
KH0149	23	1601103	231944	1600885	38.68	122711	80315	5	8
KH0153	33	1613034	218822	1612669	38.69	85421	66092	6	11
KH0155	456	3212724	68300	3189228	38.75	17428	8814	55	119
KH0156	32	1579158	311963	1577421	38.82	112821	69917	5	9
KH0158	30	1625475	181849	1625219	38.7	109177	66214	6	11
KH0159	40	1603585	233870	1602519	39	135807	74362	5	9
KH0161	74	1644954	235400	1644182	38.72	67393	36270	8	16
KH0165	42	1622199	271021	1621249	38.87	110260	63976	5	10
KH0167	42	1608729	221338	1607403	38.62	88184	50315	6	12
KH0172	40	1616334	405330	1615788	38.9	72724	52465	6	12
KH0173	28	1537605	226013	1536563	38.95	92887	48966	6	12
KH0175	37	1585432	252265	1585073	38.73	85785	55003	5	10
KH0176	61	1637534	157617	1633004	38.8	80693	43177	8	15
KH0178	45	1593565	409473	1592066	38.99	69095	47089	6	13
KH0182	44	1576421	226485	1575966	38.77	69092	48088	7	14
KH0191	30	1606355	237285	1606355	38.72	109526	77694	5	9
KH0195	667	3194887	51573	3186475	38.8	13542	5877	68	161
KH0198	99	1607611	137129	1603843	39.02	43646	28683	11	23
KH0207	54	1646883	239031	1646023	38.8	83123	58626	7	13
KH0210	49	1591902	162846	1589547	38.96	69940	51275	9	15

[a] indicates the number resulting from all contig; [b] indicates the number resulting from contigs with length > 500 bp.

Table S2. The characteristic of plasmids in *H. pylori* clinical isolates from Cambodia.

Species	Number of Plasmid	Plasmid	Accession Number	Strains Harboring the Plasmid in Cambodia
Helicobacter pylori	41	pHP489	NC_001843.1	0
		pAL202	NC_005917.1	0
		pHP51	NC_004767.1	0
		pAL236-11	NC_014161.1	0
		pHPM186	NC_001476.1	0
		pHPM180	NC_001756.1	0
		pHPO100	NC_002110.1	0
		pHPAG1	NC_008087.1	0
		pHPG27	NC_011334.1	0
		pHPP12	NC_011499.1	0
		pHPSAT464	NC_017356.1	0
		pHPv225d	NC_017383.1	0
		pHPPC4	NC_014556.1	0
		pHPLithuania75	NC_017363.1	0
		pHPSouthAfrica7	NC_017373.1	0
		pHPGambia94/24	NC_017364.1	0
		pHPPN120	NC_017377.1	0
		pHPSNT	NC_017380.1	0
		pHPF30	NC_017369.1	0
		pHPB14	NC_017734.1	0
		pXZ274	NC_017919.1	0
		pHPF32	NC_017370.1	0
		pHPELS37	NC_017064.1	0
		p1HPAKL117	NC_019561.1	0
		p2HPAKL117	NC_019562.1	0
		p1HPAKL86	NC_019564.1	0
		p2HPAKL86	NC_019565.1	0
		pHP7C	NZ_CP012906.1	0
		pHPML3	NZ_AP014713.1	0
		pHPB8	NC_014257.1	0
		pHPOK310	NC_020556.1	0
		pHPYLPMSS1	NZ_CP018824.1	0
		pHPLYSS1	NZ_CP009260.1	0

		pHPM8	NC_004845.1	0
		pHel5	NC_004949.1	0
		pHel4	NC_004950.1	0
		pHP69	NC_010884.1	0
		pHP666	NC_010932.1	0
		pAL226	NC_013547.1	0
		pAL236-2	NC_014162.1	0
		pAL236-5	NC_014163.1	0
Other	8473	other *	other *	0

* the name of plasmid and the accession number of the other species apart from *Helicobacter pylori* could be accessed in http://bioinfo.ut.ee/plasmidseeker/.

Table S3. The nucleotide variants found in 23S rRNA by comparing between 40 CLR-susceptible and 13 CLR-resistant strains.

Variants	Number of Resistant Strains		Number of Sensitive Strains		Frequency Difference (%)	*p*-Value
	Variants	No Variants	Variants	No Variants		
C14T	1	12	2	38	2.7	0.6
G15A	1	12	2	38	2.7	0.6
C39T	1	12	0	40	7.7	0.2
A60G	13	0	40	0	0.0	1.0
A181G	1	12	0	40	7.7	0.2
C183T	1	12	0	40	7.7	0.2
A229G	3	10	9	31	0.6	0.6
−239^240T	4	9	16	24	−9.2	0.8
A241T	5	8	20	20	−11.5	0.9
AATT241..244TTAA	1	12	4	36	−2.3	0.8
−242^243T	1	12	6	34	−7.3	0.9
TT243..244AA	5	8	20	20	−11.5	0.9
TTT243..245AAC	1	12	4	36	−2.3	0.8
TTT243..245C	1	12	3	37	0.2	0.7
−244^245A	1	12	2	38	2.7	0.6
A250G	1	12	0	40	7.7	0.2
A336G	1	12	6	34	−7.3	0.9
AG384..385CA	1	12	1	39	5.2	0.4
G387A	4	9	8	32	10.8	0.3
A405G	1	12	0	40	7.7	0.2
C457T	5	8	19	21	−9.0	0.8
T459C	1	12	5	35	−4.8	0.8
G469T	1	12	10	30	−17.3	1.0
A494G	4	9	3	37	23.3	0.1
T510C	9	4	36	4	−20.8	1.0
A540C	1	12	2	38	2.7	0.6
GG721..722AA	1	12	0	40	7.7	0.2
G722A	9	4	21	19	16.7	0.2
G759-	9	4	32	8	−10.8	0.9
G760A	4	9	3	37	23.3	0.1
T761A	9	4	31	9	−8.3	0.8
TTT761..763AAC	7	6	20	20	3.8	0.5

T763C	8	5	31	9	−16.0	0.9
A825G	1	12	0	40	7.7	0.2
T896C	12	1	40	0	−7.7	1.0
T976C/G/A	12	1	34	6	7.3	0.7
G1026A	3	10	10	30	−1.9	0.7
G1130A	1	12	0	40	7.7	0.2
A1282T	2	11	4	36	5.4	0.5
C1516-	9	4	25	15	6.7	0.5
CA1516..1517G	4	9	7	33	13.3	0.3
−1528^1529G	1	12	0	40	7.7	0.2
G1533A	1	12	0	40	7.7	0.2
GT1567..1568TA	5	8	5	35	26.0	0.1
T1568C	5	8	19	21	−9.0	0.8
C1633T	1	12	0	40	7.7	0.2
C1648T	7	6	24	16	−6.2	0.8
A1652G	2	11	0	40	15.4	0.1
C1699T	1	12	0	40	7.7	0.2
G1825A	1	12	3	37	0.2	0.7
A1830G	3	10	6	34	8.1	0.4
C1834T	1	12	3	37	0.2	0.7
C1955T	1	12	2	38	2.7	0.6
C1957T	1	12	0	40	7.7	0.2
A2146G	3	10	0	40	15.4	0.01221
A2147G	8	5	0	40	61.5	0.000001
C2186T	6	7	21	19	−6.3	0.8
T2199C	13	0	40	0	0.0	1.0
A2227G	2	11	7	33	−2.1	0.7
C2292T	2	11	2	38	10.4	0.2
T2299C	1	12	1	39	5.2	0.4
C2764T	1	12	5	35	−4.8	0.8
A2868G	4	9	12	28	0.8	0.6
A2921G	4	9	12	28	0.8	0.6
C2924T	1	12	4	36	−2.3	0.8
C2926T	1	12	1	39	5.2	0.4
T2929C	2	11	11	29	−12.1	0.9

Table S4. The amino acid variants in gyrA gene by comparing between 17 LVX-susceptible and 36 LVX-resistant strains.

Variants	Number of Resistant Strains		Number of Sensitive Strains		Frequency Difference (%)	*p*-Value
	Variants	No Variants	Variants	No Variants		
N4R	1	35	0	17	2.8	1.00
S5L	23	13	8	9	16.8	0.37
V6I	19	17	7	10	11.6	0.56
T9I	1	35	1	16	−3.1	0.54
N11S	1	35	0	17	2.8	1.00
G16R	1	35	0	17	2.8	1.00
G35R	2	34	0	17	5.6	1.00
V65I	1	35	0	17	2.8	1.00
A66T	1	35	0	17	2.8	1.00
N87K	12	24	0	17	32.4	0.005
A88S	1	35	0	17	2.8	1.00
D91Y/N/G	20	16	0	17	55.6	0.00004
R130K	3	33	1	16	2.5	1.00
V172I	3	33	1	16	2.5	1.00
N176D	2	34	0	17	5.6	1.00
P188S	1	35	1	16	−3.1	0.54
M191I	32	4	16	1	−5.2	1.00
D192G	1	35	0	17	2.8	1.00
D196G	1	35	0	17	2.8	1.00
V199M/A	13	23	9	8	−16.8	0.37
A207T	1	35	1	16	−3.1	0.54
G208R/E/A	23	13	10	7	5.1	0.76
D210N	1	35	0	17	2.8	1.00
E214K	1	35	0	17	2.8	1.00
A231T	6	30	3	14	−1.0	1.00
I234V	7	29	3	14	1.8	1.00
T239M	5	31	3	14	−3.8	0.70
R243H	2	34	0	17	5.6	1.00
A283V	3	33	0	17	8.3	0.54
I307V	1	35	1	16	−3.1	0.54
M314I	2	34	0	17	5.6	1.00
H354R	33	3	15	2	3.4	0.65

R367C	1	35	0	17	2.8	1.00
A379T/V	3	33	2	15	−3.5	0.65
R397Q	21	15	9	8	5.4	0.77
Q403P	10	26	3	14	10.1	0.51
P405L	1	35	0	17	2.8	1.00
A411T	9	27	3	14	7.4	0.73
M413I	1	35	1	16	−3.1	0.54
T417S	1	35	1	16	−3.1	0.54
L450R	1	35	1	16	−3.1	0.54
D454S/A	27	9	8	9	27.9	0.06
D455N	1	35	1	16	−3.1	0.54
G458S	3	33	1	16	2.5	1.00
D464E	3	33	1	16	2.5	1.00
R465H	1	35	0	17	2.8	1.00
G468K/E	28	8	13	4	1.3	1.00
T472I	1	35	1	16	−3.1	0.54
V477I	1	35	1	16	−3.1	0.54
E479G	3	33	0	17	8.3	0.54
P484Q	17	19	9	8	−5.7	0.77
R486C	3	33	3	14	−9.3	0.37
S492A/V	17	19	7	10	6.0	0.77
N495S	6	30	3	14	−1.0	1.00
I498T	4	32	2	15	−0.7	1.00
A503P	5	31	1	16	8.0	0.65
Y513H	4	32	2	15	−0.7	1.00
A524V	5	31	3	14	−3.8	0.70
K527R	20	16	8	9	8.5	0.77
S539N	21	15	7	10	17.2	0.38
E542K	1	35	0	17	2.8	1.00
D543E	1	35	0	17	2.8	1.00
H569R	5	31	0	17	13.9	0.16
I587V	13	23	5	12	6.7	0.76
A594V	1	35	0	17	2.8	1.00
D610N	4	32	0	17	11.1	0.29
A615T	1	35	0	17	2.8	1.00
N620K	7	29	1	16	13.6	0.41

S629R	1	35	1	16	−3.1	0.54
E632G	27	9	15	2	−13.2	0.47
E632_S633delinsGG	5	31	6	11	−21.4	0.14
R635fs	4	32	0	17	11.1	0.29
S636fs	4	32	0	17	11.1	0.29
C637N	4	32	0	17	11.1	0.29
C637delinsYS	20	16	14	3	−26.8	0.07
I639V/M	5	31	0	17	13.9	0.16
R640K	2	34	1	16	−0.3	1.00
G647S	1	35	0	17	2.8	1.00
S652G/N	10	26	3	14	10.1	0.51
V655I	16	20	8	9	−2.6	1.00
H668Y	28	8	17	0	−16.3	0.24
E679D	34	2	17	0	−5.6	1.00
V680A	1	35	0	17	2.8	1.00
R681C	1	35	0	17	2.8	1.00
I683M	9	27	2	15	13.2	0.47
T686V/N/S	19	17	5	12	23.4	0.14
T687A	9	27	4	13	1.5	1.00
I691R	9	27	5	12	−5.4	0.75
K694R	36	0	17	0	0.0	1.00
N696A	1	35	0	17	2.8	1.00
E697K	1	35	1	16	−3.1	0.54
V702I	4	32	0	17	11.1	0.29
G703S	5	31	2	15	2.1	1.00
S708N	18	18	7	10	8.2	0.57
D709G	6	30	1	16	10.8	0.41
G711S	12	24	6	11	−2.0	1.00
Q725R	1	35	0	17	2.8	1.00
G733E	36	0	17	0	0.0	1.00
V741I	20	16	8	9	8.5	0.77
G755S	14	22	8	9	−8.2	0.77
L766F	1	35	1	16	−3.1	0.54
A773T	1	35	0	17	2.8	1.00
R784K	13	23	8	9	−10.9	0.55
T786_G787insI	13	23	8	9	−10.9	0.55

G787R	6	30	4	13	−6.9	0.71
N789_A790delinsKT	1	35	0	17	2.8	1.00
A790T	13	23	8	9	−10.9	0.55
N797D	13	23	7	10	−5.1	0.77
M803V/I	21	15	14	3	−24.1	0.12
M803I	1	35	1	16	−3.1	0.54
V805A	13	23	9	8	−16.8	0.37
E813_P814insE	1	35	0	17	2.8	1.00
E813del	4	32	0	17	11.1	0.29
P814S/L	6	30	0	17	16.7	0.16
N816K	4	32	0	17	11.1	0.29
L817S	3	33	1	16	2.5	1.00
T819I	5	31	1	16	8.0	0.65
T819N	16	20	8	9	−2.7	1.00
S820fs	4	32	2	15	−0.7	1.00
S820P	2	34	2	15	−6.2	0.59
S821Q/P	12	24	3	14	15.6	0.33
A822T/V	8	28	2	15	10.4	0.47
A822fs	4	32	3	14	−6.5	0.67
Q823H	4	32	3	14	−6.5	0.67
N824fs	3	33	0	17	8.3	0.54
N824del	7	29	3	14	1.8	1.00
L825fs	4	32	3	14	−6.5	0.67
E827K	2	34	1	16	−0.3	1.00

Table S5. The amino acid variants in *pbp1* by comparing between 48 AMX-susceptible and 5 AMX-resistant strains.

Variants	Number of Resistant Strains		Number of Sensitive strains		Frequency Difference (%)	*p*-Value
	Variants	No Variants	Variants	No Variants		
V16I	1	4	13	35	−7.1	0.80
M17V/I	5	0	40	8	16.7	1.00
T30S	1	4	16	32	−13.3	0.87
D32N	1	4	15	33	−11.3	0.85
G44S	4	1	16	32	46.7	0.06
I79V	1	4	12	36	−5.0	0.77
K107R	1	4	7	41	5.4	0.57
P125L	5	0	46	2	4.2	1.00
I148L	1	4	21	27	−23.8	0.94
T197A	2	3	10	38	19.2	0.32
P217L	1	4	0	48	20.0	0.09
S237P	1	4	0	48	20.0	0.09
L240P	2	3	5	43	29.6	0.12
G242S	4	1	24	24	30.0	0.21
T256A	1	4	0	48	20.0	0.09
I259T	1	4	13	35	−7.1	0.80
D324N	1	4	6	42	7.5	0.52
P366L	1	4	0	48	20.0	0.09
V374L	1	4	0	48	20.0	0.09
E406K	3	2	7	41	45.4	0.041
K464_D465insK	1	4	0	48	20.0	0.09
V469M	1	4	0	48	20.0	0.09
V469A	1	4	1	47	17.9	0.18
P473L	3	2	0	48	60.0	0.0004
D479E	3	2	35	13	−12.9	0.87
N504D	4	1	40	8	16.7	1.00
D508N/E	3	2	16	32	26.7	0.33
V509I	1	4	11	37	−2.9	0.74
M515I	4	1	27	21	23.8	0.30
D535N	5	0	45	3	6.3	0.74
S543N	1	4	7	41	5.4	0.57
I563V	1	4	5	43	9.6	0.47

S589G	5	0	31	17	35.4	0.13
T593A/G/K	4	1	7	41	65.4	0.005
G595S	3	2	8	40	43.3	0.05
G595fs	1	4	0	48	20.0	0.09
V596fs	1	4	11	37	−2.9	0.74
A599T	1	4	0	48	20.0	0.09
R627H	1	4	0	48	20.0	0.09
K648fs	2	3	14	34	10.8	0.48
K648Q	1	4	6	42	7.5	0.52
R649fs	2	3	17	31	4.6	0.60
R649K	1	4	8	40	3.3	0.62
S653G	2	3	15	33	8.8	0.52
R656P	2	3	21	27	−3.8	0.73

Table S6. The amino acid variants in rdxA of each 51 MTZ-resistant and 2 MTZ-susceptible strains against 26695 reference genome.

Strains	Resistance Pattern	Mutations	Type of Mutation
KH0001	R	D59fs, K60*	frameshift
KH0012	R	R16C, T31E, Q50*	nonsense
KH0016	R	R16C, T31E, H53R, D59N, L62V, G98S, R131K, V172I, R191fs	frameshift
KH0022	R	T31E, H53R, D59N, L62V, S88P, H97T, R131K, V172I, K201fs	frameshift
KH0025	R	Q11*	nonsense
KH0031	R	R16H, T31E, H53R, D59N, L62V, A68V, S79N, S88P, G98S, R131K, V172I	missense
KH0032	R	T31E, H53R, D59N, L62V, A82V, S88P, G98S, R131K, G145W, V172I	missense
KH0033	R	R16H, T31E, D59N, K64N, P106T, R131K, P166A, V172I	missense
KH0040	R	Q6H, T49M, Q50*	nonsense
KH0042	R	T31E, Q50*	nonsense
KH0045	R	T31E, D59N, K64N, G98S, R131K	missense
KH0054	R	R16H, T31E, D59N, K64N, H97T, P106T, R131K, V204I, D205A	missense
KH0065	R	T31E, H53R, D59N, L62V, S88P, G98S, V111A, C148fs, V151*	frameshift
KH0066	R	R16C, T31E, H53R, D59N, L62V, S88P, G98S, R131K, L137*	nonsense
KH0070	R	T31E, D59N, K64N, V85fs, L89*	frameshift
KH0071	R	R16H, T31E, D59E, L62V, A68V, P106T, R131K, V172I	missense
KH0072	R	T31E, D59N, K64N, S92fs, V113*	frameshift
KH0075	R	T31E, H53R, D59E, L62V, Q65fs, N73*	frameshift
KH0086	R	R16C, T31E, H53R, D59E, L62V, S79N, S88P, P91S, G98S, R131K, G155E, V172I	missense
KH0092	S	R16H, T31E, D59N, K64N, H97T, P106S, V111A, V172I, V204I, D205A	missense
KH0095	R	S30N, T31E, H53R, D59N, L62V, S88P, G98S, Q102*	nonsense
KH0097	R	T31E, Q50*	nonsense
KH0106	R	Q6H, E35del, I36del, A37del, D59N, K60R, S88P, G98S, R131K, V172I, V204I, D205A	missense
KH0110	R	D5N, T31E, H53R, D59N, L62V, A67V, S88P, G98S, M101R, R131K, V172I, A206T	missense
KH0113	R	T31E, H53R, D59N, S88P, G98S, R131K, V172I, E175*	nonsense
KH0119	S	R16C, D59N, L62V, A118T, R131K, E175Q	missense
KH0120	R	R16H, T31K, D59N, K64N, G98S, A118T, R131K, V204I, D205A	missense
KH0122	R	Q6H, R9fs, L13*	frameshift
KH0125	R	Y26C, D59N, A68V, E75*	nonsense
KH0126	R	Y26C, E35*	nonsense
KH0127	R	S30R, D59N, K64N, Q65*	nonsense
KH0129	R	D59N, S81L, R90K, G98S, R131K, V172I, V204I, D205A	missense
KH0141	R	D59N, K64fs, M76*	frameshift

KH0143	R	R16H, T31E, D59N, A80T, S88P, H97T, P106S, R131K, V204I, D205A	missense
KH0148	R	T31E, H53R, D59N, S88P, L62V, S88P, G98S, P106S, Q130K, R131K, G170S, V172I, A206T, W209*	nonsense
KH0149	R	R9fs, D23*	frameshift
KH0153	R	R16H, T31E, H53R, D59N, S88P, G98S, R131K, V172I, K190fs, V204*	frameshift
KH0156	R	D59N, K64N, S88P, G98S, R131K, V172I, V204I, D205A	missense
KH0158	R	T31E, D59N, A82V, S88P, G98S, R131K, V172I, A206T	missense
KH0159	R	T31E, D59N, A68V, R90K, L104P	missense
KH0161	R	Q6H, R16C, D59N, H97Y, R131K, V172I, V204I, D205A	missense
KH0165	R	D59N, K64N, E75*	nonsense
KH0167	R	C19R, T31E, H53R, D59N, L62V, S88P, G98S, A118S, R131K, V172I, A206T	missense
KH0172	R	T31E, D59N, K64N, G98S, R131K, V192fs	frameshift
KH0173	R	T31E, H53R, L62V, A68V, S88P, S92fs, V113*	frameshift
KH0175	R	R16H, T31E, H53R, D59N, L62V, A68V, S88P, G98S, R131K, G162fs, D165*	frameshift
KH0176	R	R16C, T31E, A37S, D59N, R90K, V172I, A183T	missense
KH0178	R	T31E, D59N, R131K, V172I, V204I, D205A, W209*	nonsense
KH0182	R	S88P, G98S, R131K, V172I, A206T	missense
KH0191	R	6Q*	nonsense
KH0198	R	T31E, N48fs, K60*	frameshift
KH0207	R	R16H, T49M, D59N, K64N, H97T, P106S, R131K, V172I, V204I, D205A	missense
KH0210	R	R16H, T31E, H53R, D59N, L62I, S88P, G98S, R131K, V172I	missense
